# Urothelial carcinoma of the graft kidney with molecular analyses: a rare case report

**DOI:** 10.1186/s13000-021-01109-z

**Published:** 2021-06-14

**Authors:** Joyce M. Chen, G. Kenneth Haines, William Lam, Asha Reddy, Meenakshi Mehrotra, Jane Houldsworth, Qiusheng Si

**Affiliations:** Department of Pathology, Molecular and Cell-Based Medicine, Icahn School of Medicine at Mount Sinai, New York, NY USA

**Keywords:** High grade papillary urothelial carcinoma, Transplant kidney, TERT, CDKN2A/CDKN2B

## Abstract

**Background:**

Malignancy after transplantation is a leading cause of death among kidney transplant recipients. However, donor-derived malignancies are rare. We report a case of a high grade papillary urothelial carcinoma arising in a transplanted kidney.

**Case presentation:**

A 62-year-old female who received a kidney transplantation more than 30 years ago presented with urinary tract infection, acute renal failure, and hydronephrosis of the transplant kidney. Anterograde nephrostogram showed a large filling defect in the lower pole of the transplant kidney and in the proximal 3–4 cm of the ureter. A biopsy from the renal pelvic mass showed a high grade urothelial carcinoma. She underwent an anterior exenteration, resection of both transplant and native kidneys and bilateral pelvic lymph node dissection. Pathologic examination showed a high grade papillary urothelial carcinoma which appeared to arise in the pelvis of the graft kidney, involve the graft ureter and native urinary bladder. The tumor had metastasized to one left obturator lymph node but spared the two native kidneys and ureters. Short tandem repeat (STR) analysis confirmed the tumor to be of donor origin. Next-generation sequencing identified amplification of TERT and loss of CDKN2A/CDKN2B in the primary tumor.

**Conclusion:**

While it is known that transplant recipients have an increased risk of urothelial carcinoma compared to the general population, the lack of the well-documented risk factors, such as older age at transplantation, BK polyomavirus infection, and prolonged post-transplantation history and dissemination of the tumor in this case shed light on the de novo tumorigenesis of the graft kidney within the host microenvironment. Amplification of Telomerase reverse transcriptase (TERT) and loss of cyclin dependent kinase inhibitor 2A/2B (CDKN2A/CDKN2B) detected in the tumor by next gene sequencing suggests that they may play an important role in this case.

## Introduction

Kidney transplantation has been established as the treatment of choice for patients with end-stage renal disease [1, 2]. With advances in immunosuppressive regimens, transplant patients have a better quality of life and a significant survival benefit compared to those continuing on dialysis. However, transplant patients have a higher risk of developing secondary malignancies post transplantation, owing to their extended life expectancy and chronic immunosuppressive status [3]. Malignancy after transplantation has become the third leading cause of death in renal transplant recipients [4, 5]. Compared to the general population, post-transplant patients have a 7-fold increased risk of developing renal cell carcinoma (RCC) in the native kidney, where it portends a significantly worse prognosis than similar tumors arising outside of the transplantation setting. Risk factors include end-stage renal disease, longer time on dialysis, and older ages at transplant. Kidney transplant recipients also have an increased risk of developing urothelial carcinoma (UC) in the bladder and the upper genitourinary tract [6, 7], which is associated with infection with the BK polyomavirus. However, donor-derived UC is rarely reported [7–15].We herein report on a high grade papillary urothelial carcinoma arising in a donor renal allograft.

## Case report

A 62-year-old female underwent living-related donor renal transplant in 1983 due to end stage kidney disease with primary renal hypertension. She was maintained on azathrothiozine and steroids. No episodes of rejection were reported post-transplant, and the transplant kidney functioned well. In 2017, the patient presented with a urinary tract infection. Medical evaluation revealed acute renal failure and hydronephrosis of the transplanted kidney. She was treated with Cefapime and urine cytology was positive for malignant cells. An anterograde nephrostogram showed a large filling defect in the lower pole of the transplant kidney as well as a filling defect in the proximal 3–4 cm of the ureter. At time of ureteroscopy, multiple biopsies from the renal pelvic mass demonstrated high grade urothelial carcinoma. Later on, the patient was found to have extensive bladder tumors; she underwent hysterectomy, bilateral salpingo-oophorectomy, nephrectomy of native kidneys and transplant kidney, cystectomy and bilateral pelvic lymph node dissections. Post operation follow-up, pelvic magnetic resonance imaging demonstrated a large liver mass which was confirmed by biopsy to be metastatic urothelial carcinoma. Immunotherapy was initiated and the liver metastasis responded well to the immunotherapy with dramatic reduction in size of liver mass. At last follow-up, the patient was well 44 months after her surgical resection.

**Fig. 1:**
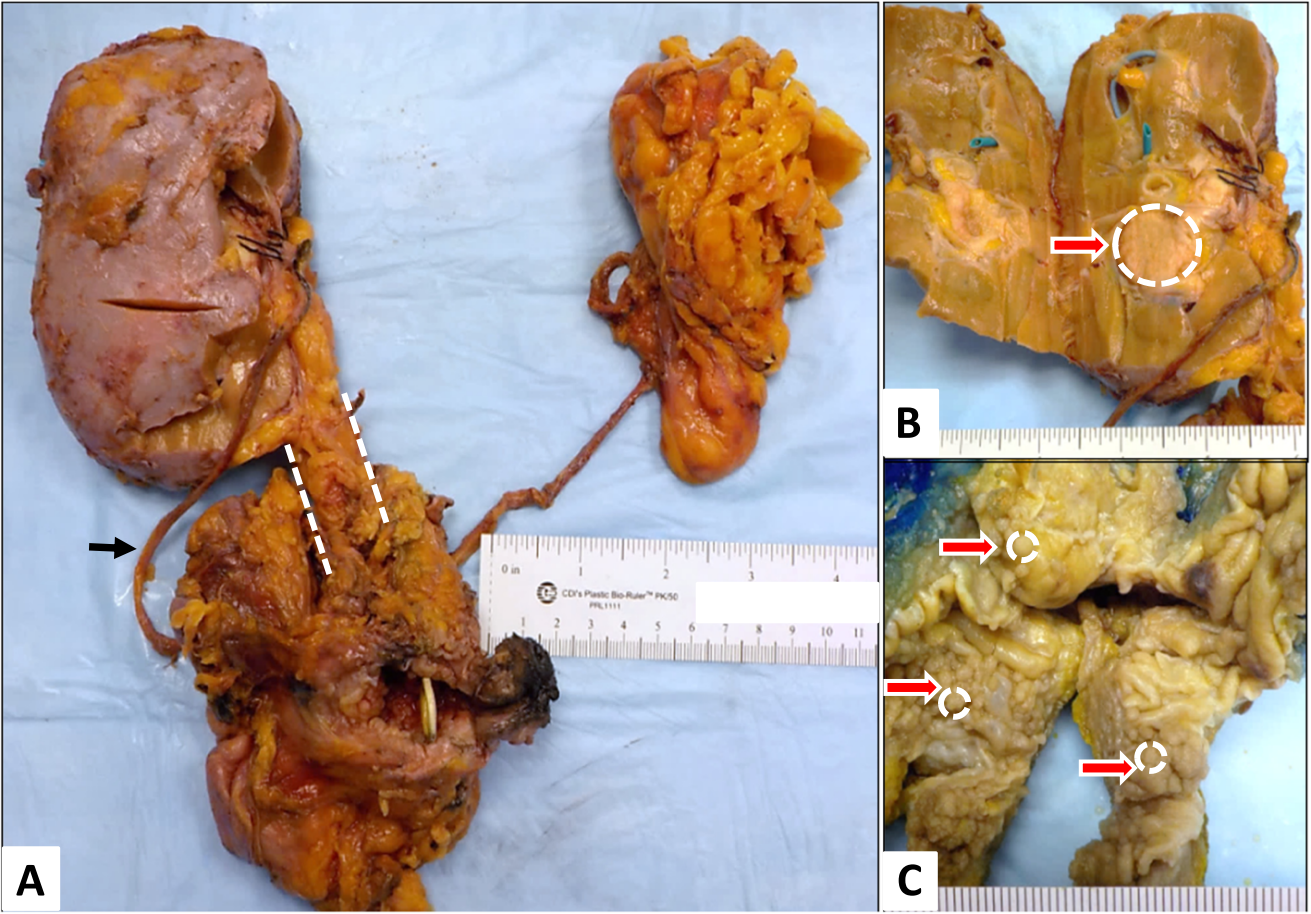
A, Gross photograph of the nephro-cystectomy specimen shows the bladder, donor kidney (left side of image) with ureter (dash line), right native ureter (black arrow), and left native kidneys (right side of image); B, Donor kidney with a renal pelvic mass; C, Native bladder with many nodules

**Fig. 2:**
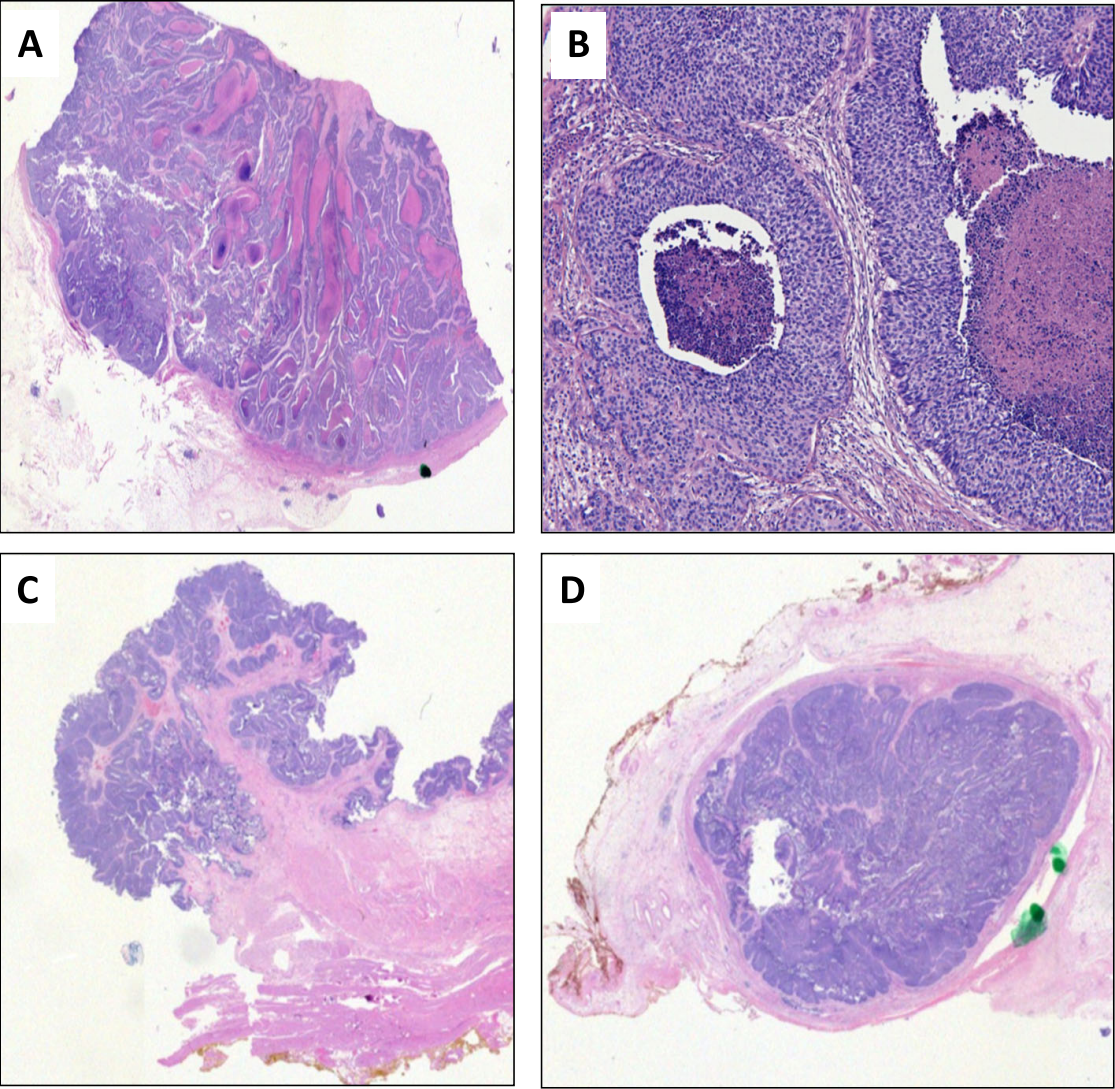
H&E stain of the high-grade papillary UC of donor kidney (A&B), urinary bladder (C) and donor ureter (D)

The Fig. 1a showed left native kidney, two native ureters, and one donor kidney connected to the native urinary bladder via a donor ureter. Upon sectioning, a 4.5 × 2.5 × 1.5 cm well-circumscribed flesh-colored mass was found in the renal pelvis of the graft kidney, a 1.2 × 1.4 × 2.5 cm flesh-colored mass encompassing the entire lumen of the proximal donor ureter, and over fifty tan discrete polypoid nodules, up to 1.5 cm, carpeting the wall of native bladder (Fig. 1b and c). The native kidneys were small (5.0 × 3.5 × 1.5 cm and 6.0 × 4.0 × 2.0 cm, respectively), with a thin cortex, poorly delineated corticomedullary junctions, and renal pelvises largely replaced by fatty tissue. No mass was identified in the native kidneys or ureters. Histology demonstrated **high grade** papillary UC involving the graft kidney, graft ureter and the native bladder. The carcinoma was predominantly non-invasive, however, focal invasion into subepithelial connective tissue was identified in the renal pelvis. Metastatic tumor was identified in one of thirteen obturator lymph nodes. The two native kidneys and ureters were negative for carcinoma (Fig. 2), demonstrating extensive thyroidization with tubular casts, calcium oxalate deposition, global and segmental glomerular sclerosis, interstitial fibrosis and chronic inflammationand arteriosclerosis. Subsequent to her surgical excision, a cystic lesion in the liver was biopsied, and confirmed to be metastatic urothelial carcinoma. The final pathological stage was therefore T1, N1, M1.

**Fig. 3:**
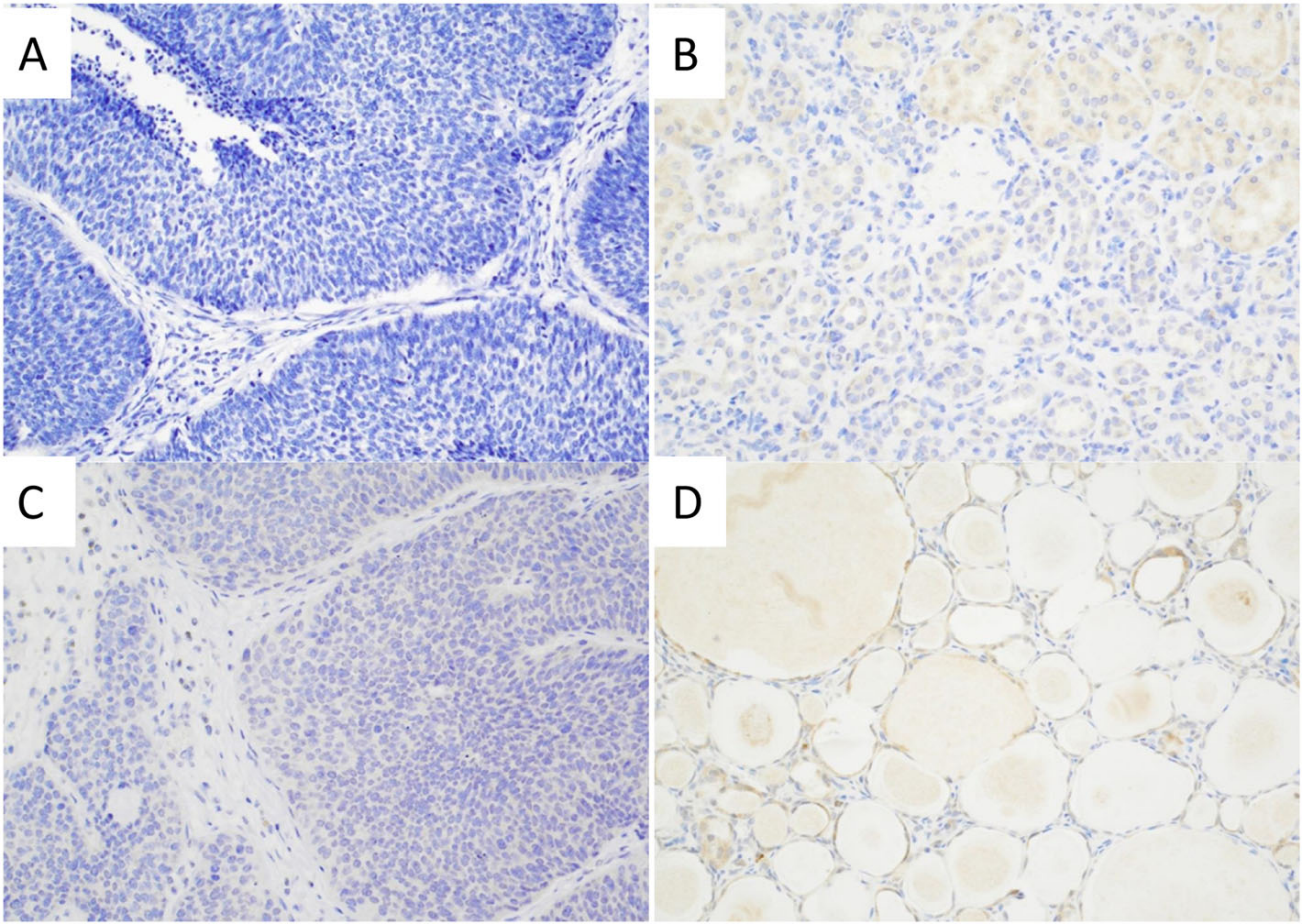
Immunostain for BK virus is negative in the UC in the renal pelvis (A), donor kidney (B), bladder (C), and in the non-neoplastic native kidney (D)

Since microsatellite instability (MSI) is commonly seen in upper urinary tract UC [16], we performed immunohistochemistry (IHC) and found that MLH1, MLH2, PMS2and MSH6 expression were retained in the UC of renal pelvis, donor ureter, and urinary bladder (not shown). BK polyomavirus has been shown as risk factors in post-transplant UC. However, both donor and recipient kidneys were negative for BK viral infection by SV40 IHC (Fig. 3). In order to determine whether the UC arose from native or donor tissue, STR analysis was performed on extracted DNA from the tumor as well as uninvolved native uterus as the control for recipient and representative apparent normal donor kidney as the control for donor. Of the 15 autosomal STRs, nine were informative. Review of these informative loci in the DNA extracted from the donor kidney tumor, ureter tumor, and bladder tumor indicated that 83, 84, and 80%, respectively, were of donor origin. These data would suggest that the tumor had drop metastasized to the bladder through the donor ureter. DNA extracted from the lymph node with evidence of metastasis showed only 8% donor origin, possibly due to low tumor cellularity in the lymph node sampled.

DNA extracted from the donor kidney tumor and the lymph node metastasis were submitted for mutation profiling of 147 genes. The results are provided in Table 1. The donor kidney tumor showed amplification of *TERT* and loss of *CDKN2A/CDKN2B,* which are genomic alterations found in approximately 7 and 25% of bladder cancers respectively (https://www.cbioportal.org). These were not detected in the lymph node metastasis. The renal pelvic tumor also showed a missense variant (p.N596S) in *MSH2* at a low VAF (3.5%). This same variant was detected in the lymph node metastasis, but at a VAF of 37%. The lymph node metastasis also showed two other missense variants in *MSH6* and *FANCA* with VAFs of 34 and 41% respectively. Interpretation of these variants would suggest these are of uncertain significance (VUS), potentially of germline origin. Given the low donor contribution in this lymph node specimen from the STR analyses (8%), it is suggested that these may be of recipient (native) germline origin. The low VAF *MSH2* detected in the donor kidney tumor confirms the 7% recipient (native) contribution detected in this specimen. Thus, for the lymph node, no apparent somatic alterations were detected, possibly due to the overall low tumor cellularity of the specimen.

**Table 1:** Mutation Profiling by Next Generation Sequencing (NGS)

Specimen	Gene	Alteration	VAF	Coverage	Significance
Donor kidneytumor (50% estimated tumor cellularity, 83% donor)					
	*MSH2*	c.1787A > G; p.N596S	3.5%	1576x	Uncertain (germline)
	*TERT*	Amplification (18.8x)			Gain-of-function
	*CDKN2A*	Loss (0.25x)			Loss-of-function
	*CDKN2B*	Loss (0.23x)			Loss-of-function
Recipient lymph node with metastasis (50% estimated tumor cellularity, 8% donor)					
	*MSH2*	c.1787A > G; p.N596S	37%	1552x	Uncertain (germline)
	*MSH6*	c.116G > A; p.G39E	34%	193x	Uncertain (germline)
	*FANCA*	c.1772G > A; p.R591Q	41%	3055x	Uncertain (germline)

## Discussion

Malignancy is a leading cause of death in renal transplant recipients. Post-transplant malignancies most often develop within the first 5 years post transplantation. Papillary RCC is the most common tumor type, almost exclusively occurring within the native kidneys [17]. These patients are also at increased risk for urothelial carcinoma, arising in the bladder and/or native kidneys [6, 7]. Donor-derived urothelial carcinoma in graft kidneys are rarely reported. Ten cases of urothelial carcinoma in grafted kidneys have been reported [7–15]. Clinical-pathologic information was not published in two of the cases. The clinicopathological characteristics of the remaining 8 patients and our case are shown in Table 2. The nine patients (5 male, 4 female) had a median age at transplantation of 46 (range 28–58) years and mean time to development of UC after transplant of 132 (range 14–408) months. The tumor from the patient with the shortest transplant to carcinoma interval arose from her third grafted kidney. In this case, the UC was proven to be of donor origin by cytogenetic analysis. Five patients presented with urinary tract related symptoms (hematuria, UTI, or flank pain). Seven of the 9 tumors were high grade carcinoma. Only one case, in addition to ours, demonstrated regional lymph node or distant metastasis. Eight of the 9 patients were treated surgically. The mean follow-up time from UC diagnosis was 28.2 (range 1.9–94) months. Only the patient with extensive tumor dissemination and without surgical treatment died shortly after diagnosis.

**Table 2:** Clinicopathological Characteristics of Urothelial Carcinoma in Graft Kidney

Reference No	Age at Transplant	Gender/ KT Type	Immuno suppressant	Initial Presentation	Age at UC Diagnosis	UC Grade	UC Stage	Interval KT to UC (month)	Treatment	F/U (Months)
[14]	41	F/DDKT	Steroid, CsA, AZA, MMF	No symptom	53	High	T3NxMx	147	NUx	Alive (94)
[10]	49	F/DDKT	Steroid, FK, MMF	Fever, Flank Pain, Urinary symptoms	61	High	T3NxMx	144	NUx	Alive (24)
[10]	57	F/DDKT	Steroid, FK, MMF	No symptom	59	High	T3NxMx	14	NUx	Alive (12)
[11]	46	M/DDKT	Steroid, CsA, AZA	No symptom	52	Low	T2NxMX	72	Partial Nephrectomy	Alive (14)
[12]	58	M/DDKT	Steroid, FK, MMF	Gross Hematuria	67	High	T2N3M1	108	CRTx	Dead (1.9)
[13]	29	M/LDKT	Steroid, CSA, MMF	Gross Hematuria	40	Low	T2NxMx	132	NUx + CRTx	Alive (24)
[8]	23	M/DDKT	N/A	Asymptomatic Microscopic Hematuria	30	High	T3NxMx	84	NUx	N/A
[9]	57	M/LDKT	FK Sirolimus	No symptom,	69	High	T3NxMx	144	NUx	Alive (12)
current case	28	F/LDKT	Steroid, AZA	UTI	62	High	T1N1M1	408	NUx	Alive (44)

*Abbreviations*: *AZA* Azathioprine, *CRTx* Chemoradiotherapy, *CsA* Cyclosporine A, *DDKT* Deceased-donor kidney transplantation, *F* Female, *F/U* Follow-up, *FK* Tacrolimus, *KT* Kidney transplantation, *LDKT* Living donor kidney transplantation, *M* Male, *MMF* Mycophenolate mofetil, *N/A* Not available, *Nux* Nephroureterectomy, *UC* Urothelial carcinoma, *UTI* Urinary tract infection

Our case of a rare donor-derived UC arising in a graft kidney is even more unusual in having arisen 34 years after transplantation, lacking other risk factors for secondary malignancy post-transplant, such as older age at transplantation and BK virus infection [18–21], and that the process spared her two native kidneys with end-stage renal disease were spared from the tumorigenesis, despite their 100-fold and 4.4-fold increased risk of developing RCC and UC, respectively, compared to the general population [3].

There are three possibilities regarding the UC origin in our case. One is that the tumor originated in the native bladder, the most common site of post-transplant UC, and spread retrograde to the donor ureter and graft kidney. The second possibility is that the primary tumor originated from the renal pelvis, and spread to the donor ureter and native bladder. This could be supported by the larger size of the tumor in the renal pelvis compared to other sites. The final possibility is the concurrent development of UC in the renal pelvis and urinary bladder. To answer these questions, we performed STR analysis, a molecular analysis of 16-STR widely used in medicine for establishing paternity and quality control in pathology. This test confirmed that the tumor in all three sites was of donor origin, supporting a renal pelvic origin, with drop metastasis to the ureter and urinary bladder.

Molecular analysis of the tumor showed a low VAF MSH2 mutation, which was not detected at the protein level by MSI IHC. This, suggests that MSI had a very limited role, if any, in this case. In contrast, amplification of telomerase reverse transcriptase (TERT) and loss of Cyclin Dependent Kinases Inhibitors (CDKN); CDKN2A/CDKN2B were detected in the tumor.

Telomerase reverse transcriptase (TERT) is the catalytic subunit of the enzyme telomerase and is essential for telomerase activity. Upregulation of TERT expression and resulting telomerase activity occurs in the large majority of malignancies, including urothelial carcinoma [22, 23]. This upregulation enables unlimited replication of cancer cells TERT activation in cancer occurs through a variety of mechanisms, including activating promoter mutations, alterations in promoter DNA methylation, chromatin remodeling, copy number alterations, and alternative splicing of TERT. Isharwal et al. [22] found 286 TERT promoter mutations and seven TERT gene amplifications in 276 tumors in a cohort of 398 UC patients. Tumors with TERT alterations had worse prognosis although, no association between TERT alterations and tumor stage or tumor grade was observed.

The CDKN2A and CDKN2B genes, encoding p16 and p15 respectively, are located on chromosome 9p21. Homozygous and heterozygous deletions occur at this locus in many primary human tumors, including urothelial carcinoma. CDKN2A and CDKN2B inhibit cyclin dependent kinase 4 (CDK4) and CDK6 which regulate cellular proliferation by preventing entry into the S phase of the cell cycle. Their inactivation may contribute to uncontrolled growth in human cancer. Deletion of CDKN2A/CDKN2B genes in urothelial carcinoma were found to be a common event and have been shown to be a crucial event in the progression from normal urothelium to carcinoma [24, 25].

Although the primary renal pelvic tumor only showed focal invasion into submucosal tissue, spread to the graft ureter, native bladder, lymph node and liver document the aggressive nature of the primary tumor. The aggressiveness in this case may be related to the patient’s immunosuppression. The role of TERT amplification and loss of CDKN2A/CDKN2B in such aggressive behavior is unknown and may require large cohort studies. Amplification of telomerase reverse transcriptase (TERT) and loss of CDKN2A/CDKN2B were not associated with UC grade and stage in tumors arising in the bladder. This may not be true in upper tract UC, as mutational differences exist between upper tract and bladder urothelial carcinomas [26].

## Conclusion

While it is known that transplant recipients have an increased risk of urothelial carcinoma compared to the general population, the lack of the well-documented risk factors, such as older age at transplantation and BK polyomavirus infection, provide an opportunity to identify possible mechanisms of tumorigenesis of the graft kidney within the host microenvironment. Amplification of TERT and loss of CDKN2A/CDKN2B detected in the tumor by next gene sequencing suggests that they may play an important role in this case.

## Data Availability

The dataset supporting the conclusions of this article is included within the article.

## Abbreviations

STR: Short tandem repeat
TERT: Telomerase reverse transcriptase
CDKN: Cyclin dependent kinase inhibitor
RCC: Renal cell carcinoma
UC: Urothelial carcinoma
MSI: **Microsatellite** instability
VAF: **Low** variant allele frequency
